# Prevalence and associated factors of adolescent undernutrition in Ethiopia: a systematic review and meta-analysis

**DOI:** 10.1186/s40795-019-0309-4

**Published:** 2019-12-09

**Authors:** Kidanemaryam Berhe, Abadi Kidanemariam, Gebrehiwot Gebremariam, Alem Gebremariam

**Affiliations:** 1 Department of Nutrition and Dietetics, School of Public Health, College of Health Sciences, Mekelle University, Mekelle, Ethiopia; 2 Department of Public Health, College of Medicine and Health Sciences, Adigrat University, Adigrat, Ethiopia; 3 Department of Nursing, College of Medicine and Health Sciences, Adigrat University, Adigrat, Ethiopia

**Keywords:** Adolescent, Undernutrition, Prevalence, Associated factors, Ethiopia

## Abstract

**Background:**

In Ethiopia, there are different pocket studies that assessed adolescent undernutrition which came up with inconsistent and inconclusive findings. Therefore, estimating the pooled prevalence and associated factors of the adolescent undernutrition using meta-analysis is crucial in Ethiopia.

**Methods:**

A systematic review of eligible articles was conducted using preferred reporting items for systematic reviews and meta-analysis (PRISMA) guidelines. A comprehensive searching of the literature was made in Pub Med, Scopus, Google, Google Scholar, Cochrane Library and CINAHL. The quality of the articles included in the review was assessed using the Newcastle-Ottawa Scale (NOS) for non-randomized studies in meta-analyses. The pooled prevalence and odds ratio of the associated factors with their 95% confidence interval was computed using STATA version 14 software.

**Results:**

Twenty-two studies were included in the meta-analysis with a total of 17,854 adolescents. Using the random-effects model analysis, the pooled prevalence of stunting and underweight was 20.7% (95% CI: 16.08, 25.33) and 27.5% (95% CI: 17.9, 57.14), respectively. Rural residence, having family size≥5, households with an unprotected water source for drinking and food-insecure household were significant associated factors for adolescent stunting. Early adolescent age (10–14 years), family size≥5, food-insecure household, lack of latrine, WHO diet diversity score < 4, mother educational status (with no formal education) were significant associated factors for adolescent underweight.

**Conclusion:**

Adolescent undernutrition remains one of the most important public health problems in Ethiopia. Almost a quarter of Ethiopian adolescents were affected by stunting and underweight. Large family size, rural residence and unprotected source of drinking water were the associated factors for adolescent stunting. Similarly, large family size, early age of adolescent, lack of latrine, low dietary diversity score, mother illiteracy, and food insecure household were the associated factors for adolescent underweight. It would be good to give high emphasis on adolescent undernutrition and it is important to address the above mentioned associated factors during adolescent nutritional interventions in Ethiopia.

## Background

An adolescent is defined as a person aged 10–19 years which is a period of gradual transition from childhood to adulthood [1]. Adolescence period is a critical period of physical growth and development. This period is very sensitive to malnutrition due to the increased physiologic need for nutrition. Adolescent malnutrition affects not only themselves but also their future generation. Underweight is low Body Mass Index (BMI) for age i.e. < − 2 standard deviation (SD). Stunting is the low height for age i.e. <−2SD. Adolescents are key to break the cycle of the intergenerational cycle of malnutrition, poverty, and food insecurity. Investing in this group of population will ensure longer-term sustainable results for reducing poverty, food insecurity, fertility, and malnutrition. However, this group has received minimal attention in the developing countries including Ethiopia. As a result, adolescent undernutrition is widespread problem prominently in economically developing countries including Ethiopia [1–5]. The prevalence of adolescent undernutrition in Ethiopia is very high and it increases over time. For example, according to the 2005 and 2011 Ethiopian demographic and health survey report, the prevalence of underweight in adolescent girls was 32 and 36%, respectively [6, 7]. In Ethiopia, overweight or obesity among adolescents is rare. Early marriage and subsequent pregnancy, lack of educational attainment, poor access to safe water and sanitation, lack of health services targeted for adolescents, lack of knowledge on the existing adolescent health services, cultures and low utilization of family planning methods were reported as a contributing factor to adolescent undernutrition. The consequences of adolescent undernutrition include delayed growth, retarded intellectual development, goiter, increase risk of infection, blindness, anemia and inadequate bone mineralization. In adolescent girls, future consequences of stunting include increased risk of adverse reproductive outcomes. E.g. risk for low birth weight, cephalo-pelvic disproportion, dystocia, and cesarean section. Again low body mass in adolescent girls is associated with reduced bone mass in early adulthood and may result in postmenopausal osteoporosis and its sequel [8–10].

Current efforts and investments in the first 1000 days of life that focuses on preventing stunting in children will be realized if there is improved adolescent nutrition. So far, most of the interventions have either focused on children aged 0–5 years or on pregnant or lactating women. However, not much attention has been paid to adolescents’ nutrition in developing countries. Today’s well-nourished adolescents will have optimal skills, talents, energies, and more of they will be healthy and responsible citizens and parents by tomorrow. To improve adolescent undernutrition and to break the intergenerational cycle of malnutrition, there should be attention and evidence-based interventions for adolescent undernutrition [11]. Notably, there are different studies which assessed adolescent undernutrition and associated factors in Ethiopia. However, they came up with inconsistency and inconclusive findings. They documented the prevalence of stunting in this group ranging from 7.2 to 40.1% [12, 13] and prevalence of underweight in this group from 5.8 to 80.8% [12, 14]. Moreover, there is no study or review that estimated the pooled prevalence of adolescent undernutrition and associated factors. Therefore, this systematic review and meta-analysis were conducted to estimate the pooled prevalence and associated factors of adolescent undernutrition, which could help policymakers and health professional for decision making.

## Methods

### Study design and search strategy

A systematic review of eligible articles was conducted using preferred reporting items for systematic reviews and meta-analysis (PRISMA) guidelines [15]. A comprehensive search of articles published in English from January 2000 through November 2017 was made from Pub Med, Scopus, Google, Google Scholar, Cochrane Library and CINAHL. In addition to these databases, the reference lists of the included studies were scanned to find potential articles. The search was performed using key terms such as adolescent undernutrition (including stunting, underweight), adolescent malnutrition, prevalence of adolescent undernutrition, magnitude of adolescent undernutrition, assessment of adolescent undernutrition, adolescent nutritional disorders, associated factors, risk factors, determinants and Ethiopia separately and/or in combination using the Boolean operator like “OR” or “AND”. Two of the authors made the search independently. Articles retrieved from the databases were exported to Endnote version X6 to facilitate the article selection process and manage citation.

### Study selection and eligibility criteria

This review and meta-analysis included studies conducted in Ethiopia with the primary objective of adolescent undernutrition and associated factors. Studies published in English and conducted at both facility and community levels were included. Studies conducted in adolescents who had diseases like HIV/AIDS, renal diseases and others were excluded. Before including the studies in the final review, they were assessed for inclusion criteria using the title, abstract and a full review of the studies. When the prevalence or associated factors were not reported, we contacted the authors, and if they did not respond or told us that the required data were not available, we excluded the study from the review. When one population was reported in more than one publication, only the most recent one or with maximum information was included in the review to avoid sample overlapping.

### Outcome measure

The primary outcome of this review was the prevalence of adolescent undernutrition i.e. stunting and underweight. According to the WHO 2006 reference data, adolescent height-for-age, and Body Mass Index (BMI) for age below − 2 Standard Deviation (SD) is stunting and underweight respectively [16]. The second outcome of this review was the associated factors of adolescent undernutrition. If one factor was identified as an associated factor for adolescent undernutrition in two and above studies (articles) then that associated factor was included in this review and meta-analysis but not considered if it identified only from one study. Age, diet diversity score (DDS), family size, household food insecurity, residence, sex and water protection were included in the analysis of associated factors for adolescent stunting. Age, diet diversity score (DDS), family size, father education status, household food insecurity, latrine, mother education status and sex were included in the analysis of associated factors for adolescent underweight.

### Quality assessment and data extraction

Two reviewers independently assessed the quality of the studies by adopting the specific protocol. The criteria proposed in the Newcastle-Ottawa Scale (NOS) for non-randomized studies were used to assess the quality of studies [17]. The following parameters were assessed: sampling strategy, inclusion/exclusion criteria, sample size, cut-offs and reference for the assessment of adolescent undernutrition status, criteria to identify undernutrition and covariates included in statistical models to identify associated factors. The final scoring system comprised 10 criteria for rating different quality elements for each study (Table 1). Any discrepancy between the two reviewers was resolved through discussion and by involving a third reviewer. A predefined data extraction format was used to collect information on the name of the author/s, year of publication, study period, study design, sample size, residence (rural, urban, both), age of study participants, sex of study participants, prevalence of stunting, prevalence of underweight and associated factors.

**Table 1:** Summary of included studies evaluating the prevalence rate and associated factors of adolescent undernutrition in Ethiopia, 2017

S.No	Author name	Year	Study design	Place of study	Region	Sex	Sample size	Prevalence of Stunting (%)	Prevalence of Underweight (%)	Quality score (out of 10)
1	Abdulkadir	2016	cross sectional	Rural & Urban	East	Both	616	17.6	33.9	10
2	Afework M	2009	cross sectional	Rural	North	Female	211	26.5	58.3	9
3	Ahmed Y	2015	cross sectional	Rural & Urban	South	Female	212	NR	13.68	7
4	Assefa H	2013	cross sectional	Rural & Urban	West	Both	2084	16	80.8	10
5	Damie	2014	cross sectional	Urban	East	Both	291	10.4	24.4	10
6	Gebregyorgis	2016	cross sectional	Urban	North	Female	807	12.2	21.4	10
7	Gebreyohanes	2014	cross sectional	Urban	Central	Both	1024	7.2	5.8	10
8	Hadush G	2015	cross sectional	Urban	North	Both	555	NR	14	7
9	Herrador	2014	cross sectional	Rural & Urban	West	Both	886	40.1	21.5	9
10	KtRoba	2016	cross sectional	Urban	South	Female	706	15.6	21.3	10
11	Mekonnen	2013	cross sectional	Rural & Urban	West	Both	790	32.2	31.2	8
12	Melaku	2015	cross sectional	Rural & Urban	North	Both	348	21.2	21.6	10
13	Meseret Y	2010	cross sectional	Rural	West	Both	425	NR	24.6	7
14	TeJI K	2016	cross sectional	Rural & Urban	Eastern	Female	546	15	21.6	9
15	TM Berheto	2015	cross sectional	Rural & Urban	South	Female	613	29.3	24.4	8
16	Wassie M	2015	cross sectional	Rural & Urban	North	Female	1281	31.5	13.6	10
17	Wolde M	2015	cross sectional	Rural & Urban	South	Both	450	NR	NR	6
18	Yebyo	2015	cross sectional	Rural & Urban	Easter	Both	411	25.5	55	8
19	MekonenT	2016	cross sectional	Urban	South	Female	598	11.9	20.9	10
20	Dessalegn A.	2017	cross-sectional	Urban	South	Both	634	NR	19.7	8
21	Yayehyirad Y	2017	cross sectional	Rural & Urban	East	Female	642	20.2	14.8	10
22	EDHS	2012	cross sectional	Rural & Urban	All (National)	Female	3724	NR	36	9

*NR* Not reported

### Publication bias and heterogeneity

To assess the existence of publication bias, funnel plots were scattered and tested for asymmetry. Egger’s test was computed [18]. A *p*-value< 0.05 was used to declare the statistical significance of publication bias. After a detailed examination of the studies by the authors, I^2^ test statistics were used to check the heterogeneity of studies. I^2^ statistics described the total variation across studies. I^2^ test statistics of < 50, 50–75% and > 75% was declared as low, moderate and high heterogeneity respectively [19].

### Statistical methods and analysis

Statistical analysis was carried out using STATA version 14. Initially, data were entered into Microsoft Excel and then exported to STATA version 14 for further analysis. The effect size of the meta-analysis was the prevalence of stunting, underweight and odds ratio of the associated factors. Random effect model was used as a method of analysis [12]. We identified associated factors for adolescent undernutrition that met the meta-analysis eligibility criteria, by looking at the adjusted ORs and 95% CIs reported in each study. Subgroup analysis was conducted by regions of the country and residence of the study participants. The effect of selected associated factors which include; Age, diet diversity score (DDS), family size, household food insecurity, residence, sex and water protection for adolescent stunting, and diet diversity score (DDS), family size, father education status, household food insecurity, latrine, mother education status and sex for adolescent underweight was analyzed using separate categories of meta-analysis. The findings of the review and meta-analysis were presented using tables, forest plots and Odds Ratio (OR) and 95% confidence intervals (CI).

## Results

### Study searches and selection

**Fig. 1:**
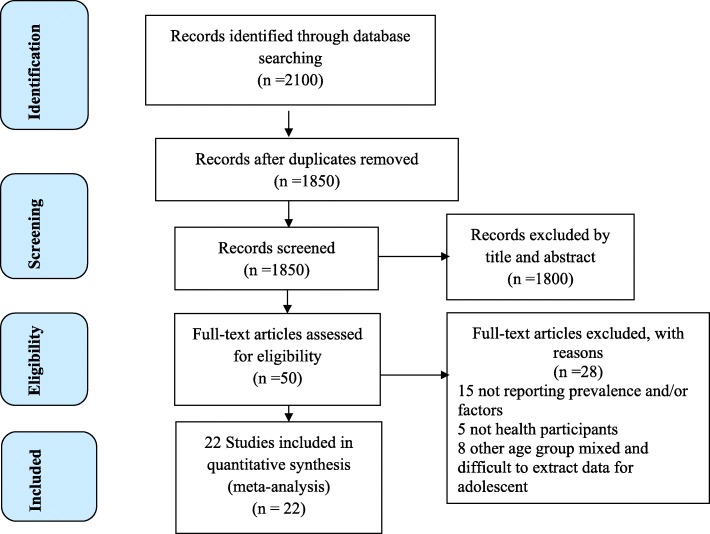
Flow diagram of the studies included in the review of adolescent undernutrition in Ethiopia, 2017

In the initial search, we found a total of 2100 records from different electronic search databases which include; Pub Med (595), Google (563), Google Scholar (490), Scopus (205), CINAHL (150) and Cochrane Library (97). From this, 250 duplicate records were removed and 1800 records were excluded after screening by title and abstracts. We assessed the full texts of 50 remaining records for eligibility, and 28 records were further excluded by the inclusion and exclusion criteria. Finally, 22 studies were considered for the final review and meta-analysis [7, 12–14, 20–37] (Fig. 1). Of the 22 studies, 16 and 21studies were used to estimate the pooled prevalence of stunting and underweight respectively.

### Characteristics of the studies and systematic review

All the studies included in this review were cross-sectional studies. A total of 17,854 adolescents were included in the analysis. The included studies reported sample size ranging from 211 [21] to 3724 [7]. Thirteen (59.1%) of the included studies were conducted in both urban and rural areas [7, 13, 14, 20, 22, 28, 30, 32–37]. One of the studies was conducted in Addis Ababa, the capital city of Ethiopia [12] and one study was conducted at the national level [7], 5 studies were from Eastern Ethiopia, 4 studies were from Western Ethiopia, 5 were from Northern Ethiopia and 6 were from Southern Ethiopia. The highest prevalence of stunting was reported from Western Ethiopia (40.1%), and the least was from central Ethiopia (Addis Ababa) (7.2%). The highest prevalence of underweight was reported from western Ethiopia (80.8%), while the least was from central Ethiopia (Addis Ababa) (5.8%) (Table 1).

### Prevalence of adolescent undernutrition

**Fig. 2:**
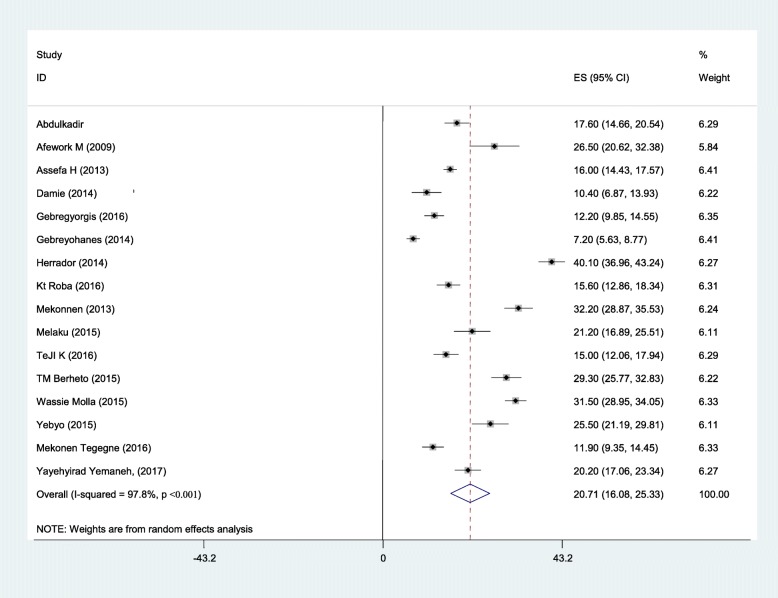
The pooled prevalence of adolescent stunting and its 95% CI in Ethiopia, 2017

**Fig. 3:**
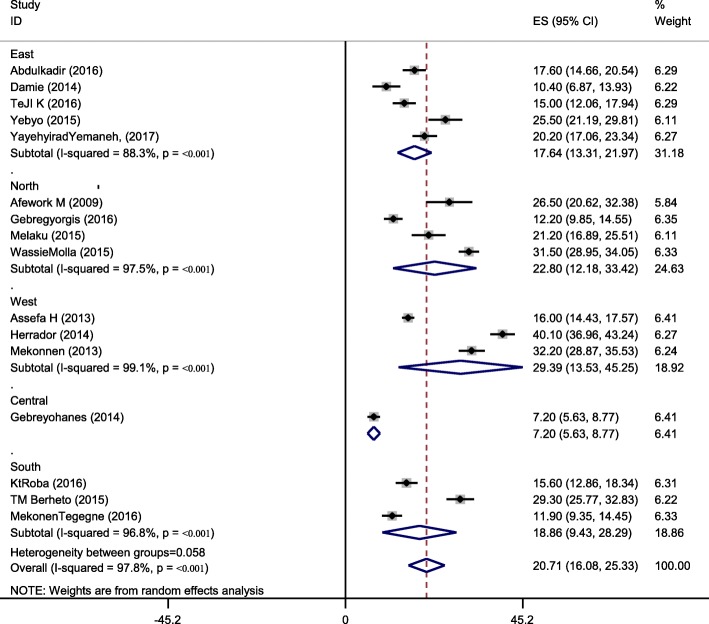
Subgroup analysis (by region) on the prevalence of adolescent stunting and its 95% CI in Ethiopia, 2017

**Fig. 4:**
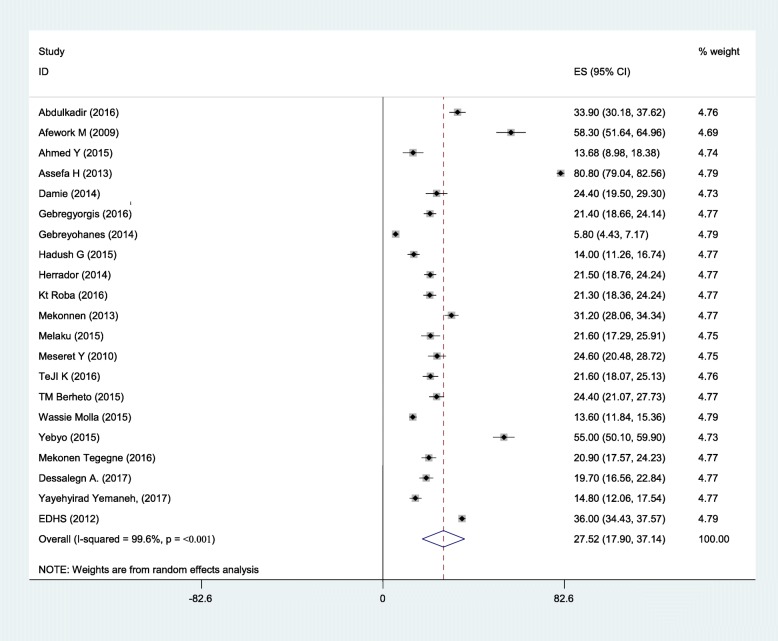
The prevalence of adolescent underweight and its 95% CI in Ethiopia, 2017

Sixteen studies were included in the analysis to estimate the pooled prevalence of adolescent stunting. The heterogeneity among the 16 studies used to estimate the pooled prevalence of adolescent stunting was very high (I^2^ = 97.3% and *p* < 0.001). Using the random-effects model, the pooled prevalence of adolescent stunting was 20.7% (95% CI: 16.08, 25.33). Twenty-one studies were included to estimate the pooled prevalence of underweight. Heterogeneity among the studies used to estimate the pooled prevalence of adolescent underweight was very high (I^2^ = 99.6% and *p* < 0.001). The pooled prevalence of adolescent underweight was 27.5% (95% CI: 17.9, 57.14). A subgroup analysis by region and residence in Ethiopia was computed to compare the prevalence of adolescent undernutrition across different areas and participant characteristics. Thus, the pooled prevalence estimated for adolescent stunting was high in western Ethiopia (29.4% (95% CI: 13.39, 45.25)), and the least was in central Ethiopia (Addis Ababa) (7.2% (95% CI: 5.63, 8.77)). Similarly, the subgroup analysis by residence showed that adolescent stunting was high in rural areas (26.5, 95%CI: 20.62, 32.38). The estimated pooled prevalence of adolescent underweight was high in western Ethiopia (44.5, 95%CI: 3.65, 85.38) and least in central Ethiopia (Addis Ababa) (5.8, 95%CI: 4.43, 7.17). Another subgroup analysis showed that adolescent underweight was high in rural areas (41.34, 95%CI: 8.32, 74.37) (Figs. 2, 3, 4 5 and 6).

**Fig. 5:**
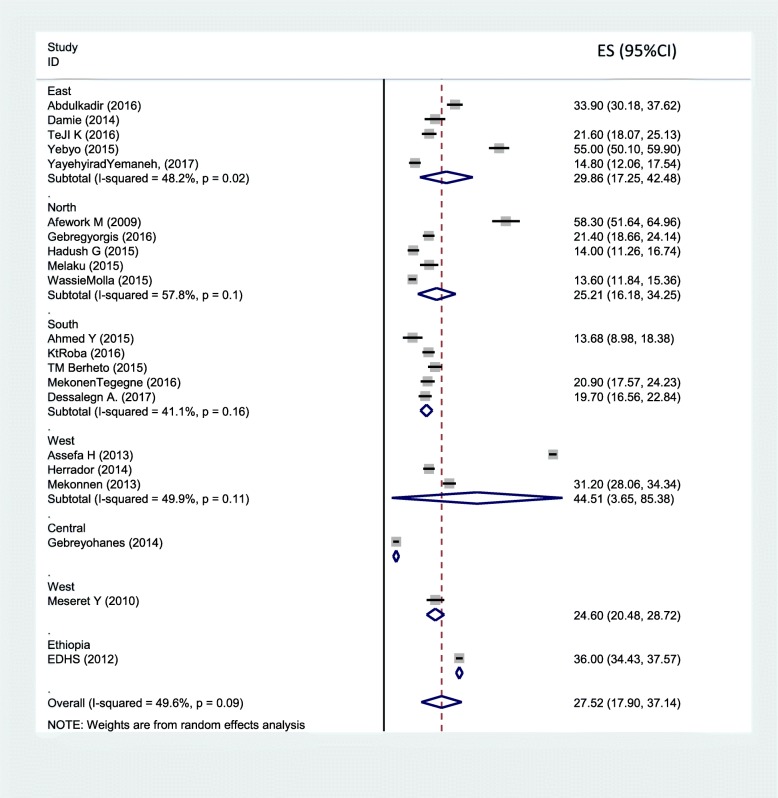
Subgroup analysis (by region) on the prevalence of adolescent underweight and its 95% CI in Ethiopia, 2017

**Fig. 6:**
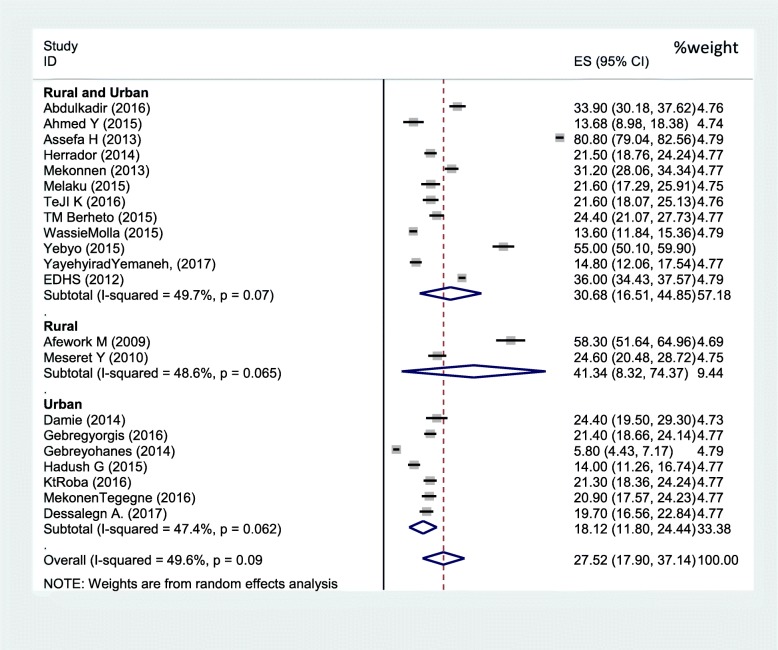
Subgroup analysis (by residence) on the prevalence of adolescent underweight and its 95% CI in Ethiopia, 2017

### The associated factor for adolescent undernutrition

Eleven studies were included in the analysis of associated factors for adolescent stunting. Six associated factors for adolescent stunting were included in the analysis. The pooled odds ratios were ranging from 0.87 to 3.39. Heterogeneity wasn’t observed among studies evaluating a residence, family size≥5, unprotected water source for drinking, and food-insecure households. From these associated factors, adolescent age and sex were not statistically significant factors for adolescent stunting but residence, family size≥5, unprotected water source for drinking and food-insecure households were statistically significant factors (Table 2).

**Table 2:** Summary of meta-analysis for associated factors of adolescent stunting in Ethiopia, 2017

s.no	Factor	Number of studies	OR (95%CI)	*P*-value	Heterogeneity			Egger’s test (*p*-value)
					Q-value	*p*-value	I^2^	
1	Residence (rural)	3	2.19 (1.59,3.02)	< 0.001	2.49	0.29	19.89	0.08
2	Age (early adolescent)	7	0.87 (0.29,2.55)	0.79	230	< 0.001	97.39	0.38
3	Family size(≥5)	2	2.25 (1.6,3.13)	< 0.001	0.44	0.51	0.000	0.16
4	Source of drinking water (unprotected)	2	3.39 (2.34,4.91)	< 0.001	0.19	0.66	0.000	0.23
5	Food insecure household	3	2.04 (1.67,2.49)	< 0.001	1.79	0.41	0.000	0.73
6	Sex (female)	5	0.99 (0.57,1.71)	0.96	22.97	< 0.001	82.59	0.84

*OR* Odds ratio, *CI* Confidence interval, Q-value = reflects study variability, I^2^ (Tua-square) = estimated variance of the observed effect sizes

Similarly, 16 studies were included in the analysis of associated factor for adolescent underweight. Eight associated factors were included in the analysis. The pooled odds ratios were ranging from 0.69 to 4.1. Heterogeneity was observed among studies evaluating adolescent age, family size, food-insecure household, sex, latrine availability, diet diversity score (DDS), and mother educational status. Thus, weights were calculated using the random-effects analysis. Early adolescent age (10–14 years)), family size≥5, lack of latrine, WHO diet diversity score < 4, mothers with no formal education were statistically significant factors for adolescent underweight (Table 3).

**Table 3:** Summary of meta-analysis for associated factors of adolescent underweight in Ethiopia, 2017

s.no	Factor	Number of studies	OR (95%CI)	*P*-value	Heterogeneity			Egger’s test (*p*-value)
					Q-value	*P*-value	I^2^	
1	Age (early adolescent)	9	2.45 (1.46,4.1)	0.001	83.14	< 0.001	90.34	0.45
2	Family size(≥5)	4	2.95 (1.76, 4.93)	< 0.001	17.62	0.001	82.97	0.69
3	Food insecure household	2	2.38 (1.54,3.69)	< 0.001	1.22	0.27	17.91	
4	Sex (female)	8	0.69 (0.41,1.12)	0.18	77.46	< 0.001	90.96	0.65
5	Lack of latrine	2	2.19 (1.09,4.4)	0.03	8.8	0.003	88.65	
6	WHO Diet diversity score (< 4)	5	1.95 (1.31,2.92)	0.001	14.39	0.006	72.2	0.88
7	Father education (illiterate)	2	0.89 (0.64,1.26)	0.5	0.93	0.34	0.000	
8	Mother education (illiterate)	5	4.1 (2.42,6.95)	< 0.001	25.59	< 0.001	84.37	0.47

*OR* Odds ratio, *CI* Confidence interval, Q-value = reflects study variability, I^2^ (Tua-square) = estimated variance of the observed effect sizes

### Publication bias

**Fig. 7:**
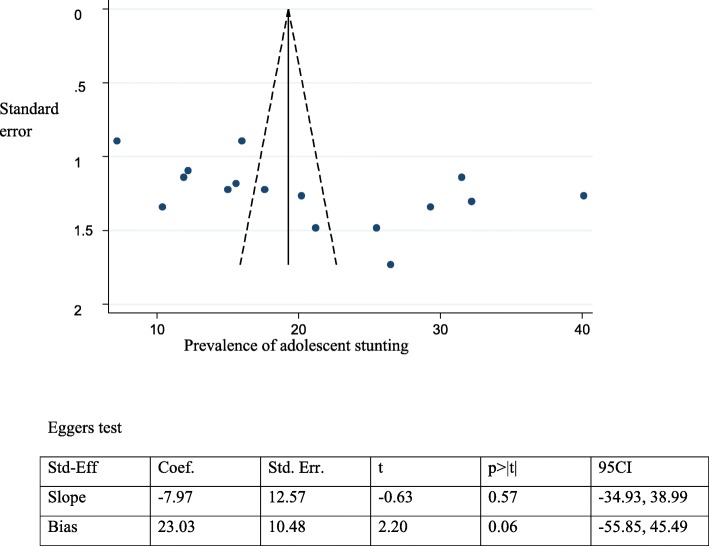
Funnel plot and Egger test to assess publication bias for adolescent stunting in Ethiopia, 2017

**Fig. 8:**
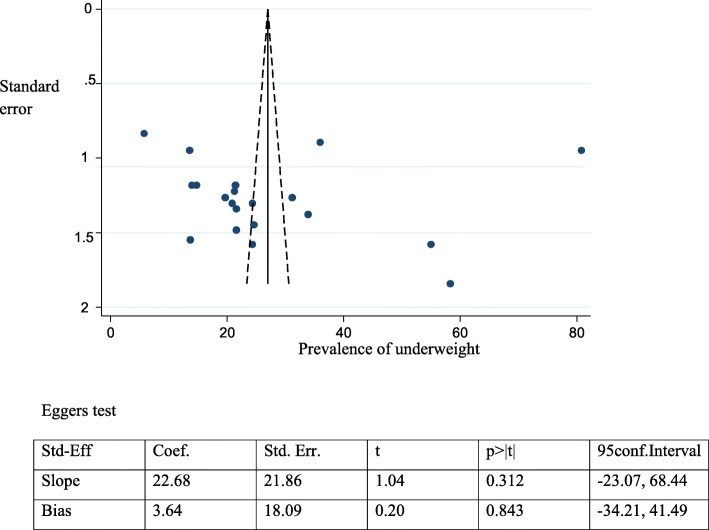
Funnel plot and Egger test to assess publication bias for adolescent underweight in Ethiopia, 2017

We assessed the funnel plot for asymmetry by visual inspection for stunting, underweight and the associated factors. The funnel plot appeared symmetrical and found no publication bias and Egger’s test was also computed for stunting, underweight and associated factors similar to the funnel plot, it revealed evidence of no publication bias (Figs. 7 and 8) (Tables 2 and 3).

## Discussion

Undernutrition negatively affects adolescents by affecting their ability to learn and work at maximum productivity, increases the risk of poor obstetric outcomes, affects sexual maturation and growth, intergenerational cycle of undernutrition, and preventing the attainment of normal bone and teeth strength [38].

This review was conducted to estimate the pooled prevalence and associated factors of adolescent undernutrition in Ethiopia. Quality of the studies included in this review was assessed using the Newcastle-Ottawa Scale (NOS) for non-randomized studies. Studies with > 5 score (out of 10 scores) were classified as good quality studies and those with ≤5 were poor quality studies. All studies had a score of > 5. In this review, the random-effects model was used for meta-analysis, considering the likelihood of significant heterogeneity amongst studies. The pooled prevalence of adolescent stunting was 20.7% (95%CI: 16.1, 25.3). Subgroup analysis using region and residence showed marked differences in the prevalence of adolescent stunting. High prevalence of stunting was observed from studies conducted in western Ethiopia and rural areas; 29.4 and 26.5% respectively. Stunting prevalence was low in central Ethiopia (Addis Ababa) and in the urban areas. The difference in the prevalence between the regions and residences could be due to the differences in socio-demographic and economic characteristics, and the number of studies included in each category of analysis. The stunting prevalence of this review is lower when we compared with the result report from studies conducted in South East Asia countries (Bangladesh (48%), Myanmar (39%)), India (54%) [39, 40] and Indonesia (23.6%) [41]. The prevalence of stunting in this review is within the range of the prevalence reported from Latin America and Caribbean countries (7–43%) [42]. The difference might be due to the difference in sampling and study period, cultural and dietary practices, access and utilization of health services.

In this review, the pooled prevalence of adolescent underweight was 27.5% (95%CI: 17.9, 37.1). Prevalence of adolescent underweight was high in western Ethiopia and in the rural areas; 44.5 and 41.3% respectively. Low prevalence of adolescent underweight was observed in central Ethiopia (Addis Ababa) and urban areas. The difference in adolescent underweight between regions as well as residences could be due to the recurrent drought and insufficient food production in western Ethiopia, especially in the rural areas. Drought, insufficient food production along with the global increased food prices can result in food insecurity and adolescent underweight [1]. According to the Ethiopia Demographic Health Survey Reports (EDHS), adolescent underweight in 2000, 2005, and 2011 was 38.4, 32.5, and 36% respectively [43]. The pooled adolescent underweight of this review (27.5%) is somewhat less than the underweight estimated in EDHS. Again underweight prevalence of this review is lower as compared to underweight prevalence studied in Bangladesh (67%), Myanmar (32%) and India (49%) [39, 40] but it is higher than the underweight prevalence reported from Latin America and Caribbean countries (3–22%), Zambia (13.7%), Nigeria (18.6%), Tanzania (21%) [42, 44–46]. The possible explanation for the differences in prevalence could be due to the differences in socioeconomic status, study period and access and utilization of health care services. Prevalence of adolescent underweight in this review is in line with the underweight prevalence reported from seven African countries (12.6–31.9%) [47].

In this review, the effect sizes of associated factors for adolescent undernutrition were estimated. The odds of having stunted adolescents were 2.25 times higher in households with a family size of ≥5 members. Likewise, a household with a family size of ≥5 members was 2.95 times higher in underweight than non-underweight adolescents. This finding is in line with a study conducted in sub-Saharan countries [48]. There is increased sharing of the available food among the large household members causing inadequate consumption of food. In addition to this, large family size usually found in uneducated parents who are more likely to accept and practice food taboo that affecting adolescent nutritional status [46]. Food insecure households were at increased risk of adolescent undernutrition. Food insecurity is one of the underline causes for undernutrition which can result in chronic nutritional problems in adolescent and cause long term negative effects in life [49].

Residence (rural) and unprotected source of drinking water were other significant associated factors for adolescent stunting. This can be explained by the inequalities in access to medical services, socio-economic status and health information in urban and rural settings. The unprotected source of drinking water is a vehicle for intestinal parasites and other communicable diseases which causes poor nutritional status. A similar finding was found in a study conducted in sub-Saharan countries [48]. Because of repeated infections, there is depressed immunity and making the severity and duration of diseases more severe and cause poor nutritional status of adolescents [41]. Age of adolescent (early age adolescent) was identified as an associated factor for adolescent underweight. There is faster growth and development in the early age of adolescent (10–14 years) as compared to late adolescent (15–19 years). Hence, if the requirement for achieving their maximum need for growth and development is not fulfilled, they will be prone to develop underweight [1]. Lack of latrine is statistically significant factor for adolescent underweight (AOR = 2.2, 95%CI: 1.1,4,4, *p*-value = 0.03). In areas where there is a scarcity of proper latrine utilization, contamination and infections will be common. Poor environmental sanitation and barefoot walking may serve as a means for parasitic infection [2, 3]. Improving dietary quality is important for increasing micronutrient intake [50]. In this review, WHO diet diversity score < 4 was higher among underweight adolescents as compared to the counterpart (AOR = 1.95 95%CI: 1.3, 2.9, *p*-value = 0.001). Low WHO DDS reflects inadequate dietary intake which can result in undernutrition [10].

Mother educational status was found as an associated factor for adolescent underweight. Educated mothers are cautious of what their family eats than uneducated mothers. Educational attainment of mother could lead to higher income and may imply a higher availability of food and household resources. It might be positively associated with higher nutritional awareness as well as better caring practices of an adolescent. Educated mothers can allocate family resources for nutrition and have health decision-making power which ultimately affects the nutritional status of the adolescent [1, 41]. This review used a comprehensive search strategy and more than two reviewers were involved in each step of the review process. PRISMA guideline was strictly followed during the review process and in order to explore the source of heterogeneity, a subgroup analysis was performed.

This review, however, has certain limitations like all the studies included were cross-sectional which could affect the temporal relationship between the assessed associated factors and outcome of interest. The number of studies for estimation of the effect size of associated factors was small which could affect the generalization of the findings. Study participants were not proportional in sex (female participants were higher in number).

## Conclusion

Adolescent undernutrition remains one of the most important public health problems in Ethiopia. Almost a quarter of Ethiopian adolescents were affected by stunting and underweight. Large family size, rural residence and unprotected source of drinking water were the associated factors for adolescent stunting. Similarly, large family size, early age of adolescent, lack of latrine, low dietary diversity score, mother illiteracy, and food insecure household were the associated factors for adolescent underweight. It would be good to give high emphasis on adolescent undernutrition and it is important to address the above mentioned associated factors during adolescent nutritional interventions in Ethiopia.

## Data Availability

All data regarding this review are contained and presented in this review document.

## Abbreviations

AOR: Adjusted Odds Ratio
CI: Confidence interval
CINAHL: Cumulative Index of Nursing and Allied Health Literature
DDS: Diet diversity score
EDHS: Ethiopian demographic and health survey
PRISMA: Preferred Reporting Items for Systematic Review and Meta-Analysis
WHO: World Health Organization
